# High Seroprevalence of Enterovirus Infections in Apes and Old World Monkeys

**DOI:** 10.3201/eid1802.111363

**Published:** 2012-02

**Authors:** Heli Harvala, Chloe L. McIntyre, Natsuko Imai, Lucy Clasper, Cyrille F. Djoko, Matthew LeBreton, Marion Vermeulen, Andrew Saville, Francisca Mutapi, Ubald Tamoufé, John Kiyang, Tafon G. Biblia, Nicholas Midzi, Takafira Mduluza, Jacques Pépin, Richard Njoum, Teemu Smura, Joseph N. Fair, Nathan D. Wolfe, Merja Roivainen, Peter Simmonds

**Affiliations:** Royal Infirmary of Edinburgh, Edinburgh, Scotland (H. Harvala);; University of Edinburgh, Edinburgh (H. Harvala, C.L. McIntyre, N. Imai, L. Clasper, F. Mutapi, P. Simmonds);; Global Viral Forecasting Initiative, Yaoundé, Cameroon (C.F. Djoko, M. LeBreton, U. Tamoufé, J.N. Fair, N.D. Wolfe);; Global Forcasting Initiatve, San Francisco, California, USA (C.F. Djoko, M. LeBreton, N.D. Wolfe);; South African National Blood Service, Weltevreden Park, South Africa (M. Vermeulen, A. Saville);; Limbe Wildlife Centre, Limbe, Cameroon (J. Kiyang);; Ape Action Africa, Yaoundé, (T.G. Biblia);; National Institute of Health Research, Harare, Zimbabwe (N. Midzi);; University of Zimbabwe, Harare (T. Mduluza);; Université de Sherbrooke, Sherbrooke, Quebec, Canada (J. Pepin);; Centre Pasteur du Cameroun, Yaoundé (R. Njoum);; National Institute for Health and Welfare, Helsinki, Finland (T. Smura, M. Roivainen)

**Keywords:** enterovirus, echovirus, seroepidemiology, nonhuman primates, apes, old world monkeys, emergence, transmission, viruses, sub-Saharan Africa, Europe

## Abstract

To estimate population exposure of apes and Old World monkeys in Africa to enteroviruses (EVs), we conducted a seroepidemiologic study of serotype-specific neutralizing antibodies against 3 EV types. Detection of species A, B, and D EVs infecting wild chimpanzees demonstrates their potential widespread circulation in primates.

Enteroviruses (EVs) form a diverse genus in the virus family *Picornaviridae*. EVs that infect humans are divided genetically into 4 species (EV A–D), and each contains numerous antigenically distinct serotypes (*1*). Although EVs were originally classified by serologic analysis and pathogenic properties in laboratory animals, sequences from the viral capsid region provide an alternative method for classification (*2*). More recently, classified variants have been assigned as chronologically numbered EV types (currently to EV-C116).

EVs also naturally infect other mammalian species, although most are in separate species from those that infect humans. However, EVs isolated from Old World monkeys (OWMs) (principally Asian macaques) are grouped into species A and B; a separate simian species (SEV-A); or are unassigned (EV-108, SV6, and EV-103) (*3*).

Although EV isolates from OWMs have been extensively characterized, little attention has been paid to EVs that circulate in apes. We recently detected EV-A76 (species A) and a new EV type in species B and D (EV-B110 and EV-D111) that infect a wild population of chimpanzees (*Pan troglodytes*) in Cameroon (*3*). Detection frequencies of 15% in fecal samples suggest that EV infections are relatively common in this species. We estimated population exposure of apes in Africa and OWM species to EVs.

## The Study

To estimate population exposure of apes and OWM species in Africa to EVs, we conducted a seroepidemiologic study of serotype-specific neutralizing antibodies against 3 EV types. These seroprevalences were compared with seroprevalences in human populations in areas where primates also lived (Cameroon, Zimbabwe, and South Africa) and with those in control populations in Europe (United Kingdom and Finland). Ethical approval for the use of study samples was obtained from the University of Zimbabwe Institutional Review Board and the Medical Research Council of Zimbabwe; the Human Research Ethics Committee, South African National Blood Service; the ethics committees of the Cameroonian Ministry of Health; the Centre Hospitalier Universitaire de Sherbrooke, Canada; and Lothian Regional Ethics Committee, Edinburgh.

EV-D94 (E210), EV-A76 (KAZ00–14550) (*4**,**5*), and a clinical isolate of echovirus 11 from Edinburgh (E-11) were used for seroprevalence studies. Neutralization assays were performed in human rhabdomyosarcoma cells as described (*6*) with 1 minor change (inactivation at 56°C for 45 min). Serum specimens at 2 dilutions (1:16 and 1:64) were incubated with virus (one hundred 50% tissue culture infectious doses) in 96-well plates. Rhabdomyosarcoma cells were added to wells (≈2 × 10^5^ cells/mL), and cultures were incubated at 37°C for <6 days. The highest dilution that completely inhibited viral replication was taken as the endpoint titer for the sample.

Plasma samples were collected from chimpanzees (*P. troglodytes*), gorillas (*Gorilla gorilla gorilla*), and several OWMs (Table 1). Sample shipments complied with the Convention on International Trade in Endangered Species of Wild Fauna and Flora. Samples were collected for veterinary welfare purposes from animals in 2 wildlife sanctuaries in Yaoundé and Limbe, Cameroon. Animals were primarily wild born and brought to sanctuaries after confiscation by authorities or abandonment by owners. Human samples were obtained from 3 sub-Saharan African populations and control groups in the United Kingdom and Finland (Table 2). None had identifiable compounding risk factors that influenced their exposure to EVs. Plasma was separated from anticoagulated blood by centrifugation and stored at −70°C until testing.

**Table 1:** Seroprevalence of echovirus and enterovirus among apes and Old World monkeys, sub-Saharan Africa and Europe

Taxonomic name (common name)	No. tested	Date	No. (%) positive		
			Echovirus 11	Enterovirus A76	Enterovirus D94
Apes					
*Pan troglodytes* (chimpanzee)	40	2005–2009	23 (57.5)	16 (40.0)	5 (12.5)
*Gorilla gorilla gorilla* (gorilla)	9	2004–2008	8 (72.0)	1 (11.0)	0
Total	49	2004–2009	31 (63.0)	17 (35.0)	5 (10.0)
Old World Monkeys					
Genus *Cercopithecus*					
*C. erythrotis* (red-eared monkey)	3	2007–2008	0	0	0
*C. preussi* (Preussi’s monkey)	3	2007–2009	0	1 (33.0)	0
*C. pogonias* (crowned monkey)	1	2009	0	0	0
*C. mona* (mona monkey)	7	2006–2009	4 (57.0)	1 (14.0)	0
*C. nictitans* (spot-nosed monkey)	3	2007–2009	0	0	0
Genus *Madrillus*					
*M. sphinx* (mandrill)	5	2006–2009	1 (20.0)	3 (60.0)	0
*M. leucophaeus* (drill)	16	2006–2009	3 (18.8)	5 (28.0)	1 (6.0)
Genus *Chlorocebus*					
*C. tantalus* (tantalus monkey)	1	2007	0	1 (100.0)	0
Genus *Erythrocebus*					
*E. patas* (patas monkey)	2	2008	0	2 (100.0)	0
Genus *Papio*					
*P. anubis* (olive baboon)	19	2006–2009	1 (5.3)	9 (47.0)	0
Total	60	2006–2009	9 (15.0)	22 (37.0)	1 (2.0)

**Table 2:** Human samples from sub-Saharan Africa and Europe tested for echovirus and enterovirus antibodies*

Country	Category	Mean age, y	Year
Cameroon	General population	>60	2007
Zimbabwe	General population	45	2007
South Africa	Blood donors	25	2009
Finland	Pregnant women	30	2002
UK	General population	55	2009

*Forty samples from each country were used.

The study was designed to determine the extent to which a human EV serotype (E-11) could spread into nonhuman populations, and conversely, the extent to which EV-A76 (previously recovered from chimpanzees) circulated in human populations in areas where chimpanzees also lived (Cameroon), elsewhere in Africa in regions without apes, and in nonprimate-exposed control populations in Europe. Species D viruses are frequently isolated from chimpanzees and gorillas (*3*), and we selected EV-D94, isolated from populations in central Africa, as a representative of this species.

Chimpanzees and gorillas showed evidence (Figure) of extensive previous exposure to E-11 (58% and 72%) and EV-A76 (40% and 11%). Lower levels of antibodies were detected against EV-D94 (13% and 0%). Conversely, OWMs showed higher seroreactivity with EV-A76 (37%) than with E-11 (15%) and EV-D94 (2%), which demonstrated wide circulation of this virus among OWM species. Seven samples from mona monkeys accounted for half of E-11–positive samples, and EV-A76 antibodies were widely distributed in baboons, mandrills, and other species.

**Figure:**
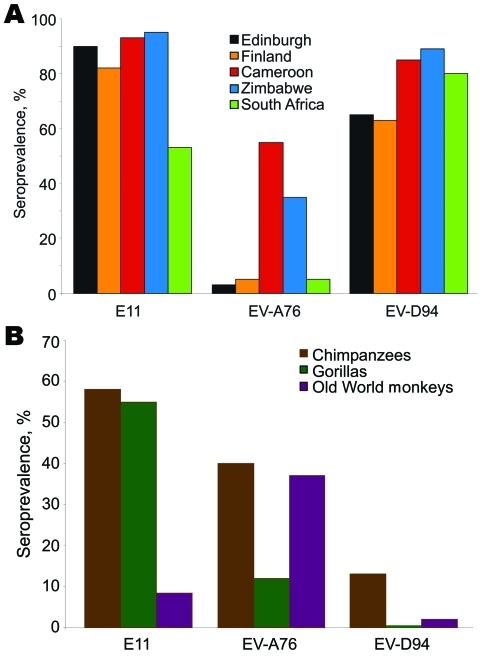
Seroprevalence of neutralizing antibody (titers >16) to echovirus 11 (E-11) and enteroviruses A76 (EV-A76) and D94 (EV-D94) in A) human populations and B) nonhuman primates.

Contrasting patterns of EV exposure to the 3 EV types were observed in humans (Figure). High seroprevalences of E-11 and EV-D94 were observed in all human populations (53%–95% and 63%–85%), whereas seroreactivity to EV-A76 was largely confined to Cameroon (55%) and Zimbabwe (35%), and uniformly <6% elsewhere.

## Conclusions

Serologic testing of nonhuman primate samples provided unequivocal evidence of exposure to all 3 serotypes. Although primate sampling was restricted to animals held in sanctuaries under veterinary supervision and infections may have been acquired in captivity from human or dietary sources, epidemiologic observations support the hypothesis that EV infections may also be acquired in the wild. Many sampled animals were adults on entry to sanctuaries, while analogous to human infections, and EV exposure is frequent during infancy or childhood. Also, EV infections in apes are widespread in the wild, and active infections are found in more than one sixth of animals screened (*3*).

Another factor determining whether EVs can be indigenous to a species or originate from repeated external (cross-species) transmission is host population size. Perpetuation of nonpersistent viruses requires minimum population sizes large enough to sustain chains of transmission, which is dependent on duration of infectivity, seasonality of infections, duration of immunity, generation time of the virus and host, rate of population turnover (*7**,**8*), and degree of fragmentation of populations. These parameters are difficult to estimate in nonhuman primates, although studies of isolated human communities show that population size needed for maintaining transmission of EVs may be large, e.g., poliovirus infections were not sustained among an Eskimo community of 450 persons (*9*). Therefore, chimpanzee and gorilla populations may be too small and fragmented to sustain EV infections. However, relatively long-term fecal excretion of EVs, the environmental stability of shed EVs, and contamination of nest sites may perpetuate infections within an established group.

In contrast to apes, the population size of several OWM species is large, and with higher population connectivity, turnover, and supply of susceptible animals, it may support indigenous circulation of EVs. Supporting this suggestion is genetic evidence that EVs isolated from OWMs in southern Asia and Africa group separately from most variants identified in humans elsewhere by phylogenetic analysis (*10*). EV variants in chimpanzees that matched OWM serotypes (e.g., EV-B110 most closely related to SA5, and EV-A76, EV-A89, and EV-A90 in the OWM species A group) (*3*; unpub. data) suggest that OWMs are a potential source of infection. Predation of the red colobus monkey by chimpanzees (*11**,**12*) may favor such cross-species transmissions, as documented in the genesis of simian immunodeficiency virus_CPZ_ from OWM simian immunodeficiency viruses (*13*). Cross-species transmission from OWMs to apes is consistent with high seroprevalences of EV-A76 antibodies in apes, baboons, and other OWMs.

Overall, our serologic survey data and previous fecal sampling data (*3*) provide evidence for extensive circulation of EVs between primates and existence of human and OWM reservoirs of infection that may spill over into ape populations too small to maintain indigenous EV variants. Whether OWMs or apes represent a potential source of new EVs in humans (that may become pandemic in the absence of prior population exposure) is uncertain. However, the global outbreak of EV-D70 that originally centered on a cluster of human infections in central Africa (*14**,**15*) provides a potential example of this occurrence. Extensive past infection of a variety of EVs in apes and OWMs should lead to a reappraisal of the host range of what have been considered to be primarily human viruses and a potential source for the periodic emergence of new EV types into immunologically naive human populations.
