# Correction: Vol. 18 No. 2

**DOI:** 10.3201/eid1805.C21805

**Published:** 2012-05

**Keywords:** Correction: Vol. 18 No. 2, Erratum, Harvala, 11-1363

Author Richard Njouom’s surname was misspelled in High Seroprevalence of Enterovirus Infections in Apes and Old World Monkeys (H. Harvala et al.). The article has been corrected online (wwwnc.cdc.gov/eid/article/18/2/11-1363_article.htm).

